# Impact of Respectfulness on Semantic Integration During Discourse Processing

**DOI:** 10.3390/bs15040448

**Published:** 2025-04-01

**Authors:** Wenjing Yu, Yuhan Xie, Xiaohong Yang

**Affiliations:** Department of Psychology, Renmin University of China, Beijing 100872, China

**Keywords:** respectfulness, semantic integration, global resource theory, language comprehension, discourse processing

## Abstract

Linguistic expressions of respectful terms are shaped by social status. Previous studies have shown respectful term usage affects online language processing. This study investigates its impact on semantic integration through three self-pace reading experiments, manipulating Respect Consistency (Respect vs. Disrespect) and Semantic Consistency (Semantic Consistent vs. Semantic Inconsistent). In Experiment 1, disrespect was manipulated by using the plain form of pronouns instead of the respectful form when addressing individuals of higher social status. The results showed longer reading times for semantically inconsistent sentences compared to consistent ones, reflecting the classic semantic integration effect. Nevertheless, this effect was only detected when respectful pronouns were employed. For Experiments 2 and 3, disrespect was operationalized by directly addressing individuals of higher social status by their personal names. A comparable interaction to that in Experiment 1 was identified solely in Experiment 3, which involved an appropriateness judgment task. In contrast, no such interaction was observed in Experiment 2, which involved a reading comprehension task. These results indicated that both disrespectful pronouns and addressing individuals by their personal names hinder semantic integration, but through different mechanisms. These findings provide important insights into the role of respectful term usage on semantic integration during discourse comprehension.

## 1. Introduction

In linguistic communication, the use of respectful terms often reflects the social relationship between the speaker and the listener. The choice of respectful terms not only follows linguistic rules but also conveys cultural connotations. In the Modern Chinese Dictionary, address terms are defined as terms people have because of kinship or other relationships as well as their status, occupation, etc., such as father, master, and boss. To be brief, terms of address are words or expressions used to indicate certain relations between people, or to show the difference in identity, position, and social status. They are reflections of national cultures. They play a very important role in face-to-face communication since it is the first information transferred to others (C. Yang, 2010). Research has shown that the selection of respectful terms significantly influences the understanding and reactions of both parties in a conversation. For instance, using more appropriate respectful terms can enhance the effectiveness and friendliness of communication (Brown & Levinson, 1987).

In hierarchical and status-oriented societies, traditional values emphasizing respect for the elderly and individuals with higher status necessitate careful consideration when choosing forms of address. For instance, older individuals should not be addressed directly by their names, a convention that applies universally, including within familial relationships such as between siblings (Leech et al., 2014). However, existing research primarily focuses on the sociopragmatic functions of respectful terms, with limited exploration into their specific impact on discourse semantic integration. Semantic integration is the process of linking newly encountered words in reading to the preceding context to form a coherent representation. This requires not only understanding individual word meanings but also selecting or constructing appropriate interpretations to integrate them into the context for coherent comprehension (Hagoort et al., 2009). Therefore, this study aims to explore how the use of respectful terms affects discourse semantic integration through three experiments.

In many languages, the use of respectful terms during verbal communication must adhere to rules that align with social status (or social distance) information (Agha, 2007). For example, in ancient China, to show respect for the emperor or ancestors, their names were never mentioned, either verbally or in writing (Lee, 2019). In Chinese culture, the concept of “respectfulness” plays a crucial role. When pronouns are used to represent the second person, there are generally two forms. One is “ni” (你 in Chinese, means “you_[plain]_”), hereafter referred to as “ni” (you_[plain]_), and the other is “nin” (您 in Chinese, means “you_[respectful]_”), hereafter referred to as “nin” (you_[respectful]_) (Leech et al., 2014). When younger generations speak to elders, they typically use the respectful pronoun “nin” (you_[respectful]_) to show respect. For example, a grandson would usually address his grandfather as “nin” (you_[respectful]_) to express respect. This practice is maintained in conversations to avoid violating social norms or causing misunderstandings (Lee-Wong, 2000; Zhou, 2008). In Korea, it is considered inappropriate to directly call someone of higher social status by their personal name (Kwon & Sturt, 2016). Chinese and Korean cultures share similarities in this regard; Chinese people also avoid addressing elders by their personal names, and characters identical or homophonous to elders’ names are often avoided as well (X. Shi, 2006). People are accustomed to using honorifics when addressing individuals of higher status (C. Yang, 2010).

Some studies have explored the influence of social status information on the use of respectful pronouns “ni” (you_[plain]_) and “nin” (you_[respectful]_). For example, Jiang et al. (2013) examined the impact of inappropriate use of these respectful pronouns (“nin” (you_[respectful]_) and “ni” (you_[plain]_)) under different social statuses (Jiang et al., 2013). The results showed that, compared to conditions of status consistency, status inconsistency (such as over-respect and disrespect) elicited a larger N400 response. Similarly, Jiang and Zhou (2015) found that participants were sensitive to social status information in conversations. In conversation evaluations, the use of pronouns consistent with social status was rated as more appropriate, while those inconsistent with social status were rated as less appropriate (Jiang & Zhou, 2015). The findings of Ji et al. (2022) indicate that different respectful pronouns (“nin”(you_[respectful]_) and ”ni” (you_[plain]_) evoke distinct ERP responses related to semantic consistency. Inappropriate use of respectful pronouns, in particular, elicits an N400 effect. These studies suggest that during reading comprehension, readers experience greater cognitive load when respectful pronouns are used inappropriately (Ji et al., 2022).

Further studies have shown that the inappropriate use of respectful pronouns not only hinders their own processing but also interferes with the integration of other information within sentences (Ji, 2021; Ji & Ji, 2023). For instance, Ji (2021) investigated the influence of politeness consistency on semantic integration by manipulating violations of respectful pronouns and word class (adjective–noun combinations). The results showed that inappropriate use of respectful pronouns elicited a P200 effect in the 190–320 ms range and a central-parietal positivity in the 360–866 ms range. When respectful pronoun and word class violations occurred simultaneously, a P600 effect was observed in the 362–868 ms window (Ji, 2021). In a follow-up study in 2023, Ji et al. grouped participants based on Autism Quotient Communication (AQ-Comm) subscale and manipulated conditions of respectful pronoun and word class violations. They found that, under word-class consistency conditions (e.g., “黄丽告诉她那好学的儿子。”/Gloss: “Huang Li told her that studious son.”/Translation: “Huang Li told her studious son.”), the inappropriate use of respectful pronouns (“你的书到了。”/Gloss: “you_[plain]_ book has arrived.”/Translation: “your book has arrived.”) elicited a P600 in the pragmatically less-skilled subgroup (as indexed by higher scores on the AQ-Comm subscale), but a left anterior negativity (LAN) and late negative component in the pragmatically skilled subgroup (as indexed by lower scores on the AQ-Comm subscale). In cases of combined violations of word class and respectful pronouns, a P600 effect was observed in both groups (Hagoort et al., 2004; Ji & Ji, 2023). In summary, these studies indicate that the appropriateness of respectful term usage affects the online processing of other linguistic information in sentences. However, the above studies mainly focused on single sentences and did not examine whether the inappropriate use of respectful terms affects semantic integration in discourse context (e.g., discourse comprehension or conversation), which limits our understanding of the role that respectful term usage plays.

Semantic integration refers to the process of integrating newly read information with previously read context to form a coherent sentence representation (Hagoort et al., 2009). Researchers typically manipulate semantic consistency in sentences or texts and compare inconsistent conditions with consistent ones to observe semantically integration effects (Y. Shi et al., 2024; Chang et al., 2020; X. Yang et al., 2020; Wu et al., 2019; Wang et al., 2011; Lassonde & O’Brien, 2013). Behavioral studies have consistently found that response times for semantic inconsistent sentences are longer than for semantically consistent sentences (Y. Shi et al., 2024; Wu et al., 2019; Lassonde & O’Brien, 2013). For instance, Y. Shi et al. (2024) manipulated verb types (e.g., “赡养”/Gloss: “support raise”/Translation: “to provide for and support (typically elderly parents)” vs. “看见”/Gloss: “look see”/Translation: “see someone”) and status matching (e.g., “grandfather” vs. “father”) to create four conditions. They found that sentences with inappropriate verbs and mismatched status (e.g., “爷爷赡养爸爸”/Gloss: “Grandfather supports Dad.”/Translation: “The grandfather carefully supported the father.”) had the longest reading times. ERP studies typically found that Semantic Inconsistent condition, compared to Semantic Consistent condition, elicited larger N400 and P600 amplitudes (X. Yang et al., 2020). This suggests that during language processing, individuals immediately integrate newly read information with the context of the sentence or discourse.

Due to the importance of semantic integration, many studies have investigated how factors such as contextual constraint (Chang et al., 2020) or information structure (Wang et al., 2011) influence semantic integration in sentences or discourses. However, no studies have yet examined how semantic integration is affected by respectful terms within a discourse context. Given the pervasive role of respectful terms in shaping social interactions and their demonstrated influence on online language processing (Jiang et al., 2013; Ji et al., 2022), this gap limits our comprehensive understanding of discourse comprehension. Investigating the interplay between respectful term usage and semantic integration is therefore critical, as it bridges linguistic processing with sociocultural norms, offering insights into how social cues modulate cognitive mechanisms during language comprehension.

In Chinese culture, respectful terms (respectful pronouns and personal name) have inherent usage norms, and people are not allowed to violate these norms. According to the global resource theory, inappropriate linguistic elements, such as disrespect demands additional cognitive resources, thereby interfering with semantic integration. In contrast, the binding theory (Christianson et al., 2017) suggests that language processing involves establishing referential relationships between linguistic elements, and disrespect may disrupt these bindings, leading to processing difficulties. Together, these theories explain how the presence of inappropriate respectful terms influences sentence processing and semantic integration. Specifically, the global resource theory posits that individuals perform worse on memory tasks involving inappropriate words. Essentially, attentional resources are allocated to inappropriate words, leaving limited processing time and causing surrounding words to be ignored (MacKay et al., 2004). Christianson et al. (2017) conducted an online sentence processing study involving inappropriate words and found that sentences containing inappropriate words required longer processing times because the inappropriate words captured participants’ attention, supporting the global resource theory (Christianson et al., 2017). The effect of inappropriate words slowing down concurrent task processing is also observed in other studies, including the attentional blink task (Mathewson et al., 2008), taboo word distractors during picture naming (Dhooge & Hartsuiker, 2011), and taboo word distractors before lexical, animacy, rhyme decision, and nonword naming tasks (Zeelenberg et al., 2011). Taken together, according to the global resource theory, processing inappropriate words requires more attentional resources, leading to processing disadvantages. Accordingly, it can be inferred that the inappropriate use of respectful terms attracts more attention from participants, leaving insufficient attentional resources for semantic integration, thus hindering semantic integration.

In contrast, the binding theory posits that inappropriate words attract attention and link stimuli to their context, leading to better memory and stronger contextual binding (Christianson et al., 2017). In other words, the attention elicited by inappropriate words facilitates better information integration, ultimately enhancing memory performance. For instance, it has been found that the presence of inappropriate words enhances memory performance (Guillet & Arndt, 2009). Studies involving tasks such as word list presentation (Hadley & MacKay, 2006; Buchanan et al., 2006; Grosser & Walsh, 1966) and repetition priming (Thomas & LaBar, 2005) have also observed enhanced memory effects for inappropriate words. Therefore, according to the binding theory, the presence of inappropriate respectful terms could strengthen the binding between sentence components, thereby improving semantic integration (Christianson et al., 2017).

To explore these two possibilities, this study will investigate the impact of the inappropriate use of respectful terms (Respect Consistency) on discourse semantic integration through three experiments. Respect Consistency refers to the alignment between the form of address used and the expected level of politeness dictated by social norms. In this study, it is operationalized by distinguishing between Respect conditions (e.g., “nin” [您]) and Disrespect conditions (e.g., “ni” [你]) in reference to individuals of higher social status. This manipulation follows the previous research on politeness strategies and language processing (e.g., Kwon & Sturt, 2016), which highlight how deviations from expected respectful forms can affect cognitive processing during discourse comprehension. In this study, Semantically Consistent and Semantically Inconsistent were manipulated by controlling the relationship between the first sentence (S1) and the fourth sentence (S4) in the text. Specifically, S1 describes an event, a person, or an object, while S4 provides additional information that may or may not align with the world knowledge conveyed by S1. When the information is aligned, it establishes a Semantically Consistent condition; when it is not, it results in a Semantically Inconsistent condition. For instance, as shown in Table 1, if S1 states that Xiaoming is 7 years old and S4 indicates that Xiaoming is doing homework, the information in S4 is consistent with that in S1, creating a Semantically Consistent condition. In contrast, if S1 states that Xiaoming is 30 years old and S4 describes Xiaoming doing homework, the information in S4 conflicts with that in S1, leading to a Semantically Inconsistent condition. This manipulation allows us to examine how deviations from expected world knowledge influence real-time sentence processing and semantic integration, following the paradigms used in previous studies on semantic violations (Y. Shi et al., 2024; Wu et al., 2019; Lassonde & O’Brien, 2013).

In Experiment 1, we will manipulate the Respect Consistency of respectful pronouns (Respect (“nin” (you_[respectful]_)) vs. Disrespect (“ni” (you_[plain]_))) and Semantic Consistency (Semantic Consistent vs. Semantic Inconsistent) to examine the effect of inappropriate use of Respect condition (“ni” (you_[plain]_)) on semantic integration. In Experiment 2, we will further manipulate the Respect Consistency of personal names (Respect (appropriate respectfulness) vs. Disrespect (personal names)) to examine the effect of personal names on semantic integration. In Experiment 3, we will replace the reading comprehension task from Experiment 2 with the appropriateness judgment task.

According to the global resource theory (MacKay et al., 2004), in Experiment 1, we predict that in the Respect condition, reading times may be longer for Semantically Inconsistent sentences than for Semantically Consistent ones. However, in the Disrespect condition, since more attentional resources are allocated to disrespectful expressions, there may be no significant reading times differences between the two semantic conditions. In Experiment 2, we expect to obtain similar results to those in Experiment 1. Experiment 3 builds on Experiment 2 by introducing a cognitively more demanding explicit task to further investigate the effect of respect on semantic integration. Hence, we anticipate that the results may resemble those of Experiment 1. In contrast, the binding theory posits that the presence of inappropriate words facilitates semantic integration (Christianson et al., 2017). Drawing on this theory, we predict consistent outcomes across all three experiments. Specifically, in the Respect condition, we expect significant reading time differences between the Semantically Consistent and Semantically Inconsistent conditions. In the Disrespect condition, however, a substantially larger and more significant difference between the two semantic conditions may emerge. Table 1 also illustrates these predictions.

## 2. Experiment 1

### 2.1. Methods

#### 2.1.1. Participants

The required sample size was calculated using G*Power 3.1 software (Faul et al., 2009) prior to the experiment. By setting the effect size as 0.25 and power (1-*β*) as 0.95 in G*power, we obtained a sample size of 36 subjects (Ji & Cai, 2022). To account for potential data loss due to participant performance issues, we recruited 48 students for the experiment. Participants’ ages ranged from 18 to 29 years (*M* = 21.67, *SD* = 2.84), including 15 males and 33 females. Ultimately, data from all 48 participants were included in the analysis. All participants were undergraduate or graduate students at Renmin University of China, long-term residents of China, and self-reported native speakers of Chinese. They passed the Putonghua Proficiency Test and scored above 120 on the Chinese section of the National College Entrance Examination, ensuring high Mandarin proficiency and minimizing dialectal influences. All were right-handed, had normal or corrected-to-normal vision, and had no history of reading disabilities or psychiatric disorders. This study was approved by the Academic Ethics Committee of the Department of Psychology at Renmin University of China. Participants provided informed consent prior to the experiment and received compensation upon completion.

#### 2.1.2. Experimental Materials

The experimental materials consisted of 80 sets of stimuli, resulting in a total of 320 passages. Each passage comprised five sentences: the first provided background information, the second introduced the respectful term, the third presented the dialogue content, the fourth was the critical sentence, and the fifth concluded the passage. The critical sentence and the first sentence formed either a Semantic Consistent or Semantic Inconsistent pair (see Table 2).

To ensure the validity of the experimental materials, 16 participants, not involved in the main experiment, were recruited to evaluate the stimuli. Using a 7-point Likert scale (1 = very unreasonable, 7 = very reasonable), participants rated both the Semantic Consistency and the Respectful Consistency. The materials were divided into four versions, with each version rated by four participants. To avoid familiarity effects, sentences from each condition appeared only once per participant. We calculated the scores for the two dimensions under different conditions. For Respectful Consistency, the scores were as follows: Respect (*M* = 6.78, *SD* = 0.23), Disrespect (*M* = 2.22, *SD* = 0.67). For Semantic Consistency, the scores were Semantic Consistent (*M* = 5.98, *SD* = 0.61), Semantic Inconsistent (*M* = 1.95, *SD* = 0.69). A paired *t*-test was conducted to analyze the evaluation results. The results showed significant differences in the appropriateness of the Respect Consistency, *t* (79) = 51.16, *p* < 0.01, indicating that participants could clearly distinguish between Respect and Disrespect. Significant differences were also found in Semantic Consistency, *t* (79) = 47.00, *p* < 0.01, suggesting that participants could clearly differentiate between Semantically Consistent and Semantically Inconsistent. These findings demonstrate that the experimental materials met the required standards of validity. Participants were able to understand the intended distinctions in Semantic Consistency and Respect Consistency, ensuring that the materials effectively captured the targeted contrasts.

We employed a 2 (Semantic Consistency: Semantically Consistent, Semantically Inconsistent) × 2 (Respect Consistency: Respect “nin” (you_[respectful]_), Disrespect “ni” (you_[plain]_)), within-subjects experimental design. The dependent variable was the reading time of the critical sentence (S4). The experiment utilized a Latin square design, and the formal experiment included 80 sets of key experimental materials, divided into four versions according to the Latin square order. Each version contained 80 critical sentences and 80 filler sentences, with each participant assigned one version. To prevent participants from detecting that the violation in the discourse always occurred in sentence 4, the filler materials included both semantically consistent passages and passages with semantically inconsistent in various positions. Specifically, the filler materials consisted of 80 items in total, with the Semantically Inconsistent pair set in the 1st, 3rd, 1st and 5th, 3rd and 4th, and 3rd and 5th sentences. Each of these conditions contained 10 items, while 30 items consisted of semantically correct sentences.

#### 2.1.3. Experimental Procedure

Participants sat in front of a computer screen, and the experimental materials were presented using E-prime 2.0. The experiment followed a self-paced reading paradigm. In each trial, a fixation point was displayed for 1000 ms, followed by the sequential presentation of five short sentences. Participants controlled the presentation speed using the spacebar, with instructions to read as quickly as possible. After reading the fifth sentence, a blank screen appeared, followed by a comprehension judgment task with a one-third probability. Within 3 s, participants were required to judge whether the sentence accurately reflected the content of the passage. If the judgment was correct, they pressed the “J” key; otherwise, they pressed the “F” key. Before the formal experiment, participants practiced with 9 sets of passages to familiarize themselves with the procedure. The formal experiment lasted approximately 30 min.

#### 2.1.4. Data Analysis

A linear mixed model (Baayen et al., 2008) was used to analyze the reading time of the critical sentence (S4). The independent variables were Respect Consistency (Respectful, Disrespectful) and Semantic Consistency (Semantic Consistent, Semantic Inconsistent), while the dependent variable was the online reading time of the critical sentence (S4). Respect Consistency, Semantic Consistency, and their interaction were treated as fixed effects, with participants and items as random effects.

Initially, we evaluated the maximal model to check for a singular fit, which could indicate that the random effect structure was overfitted (Paul et al., 2021; Stenbäck et al., 2021). If such issues were encountered, we removed one random slope based on correlation estimates close to −1 or 1, thereby reducing the random effect structure and achieving a non-singular fit (Barr et al., 2013). After each modification, we re-assessed the model’s fit. If convergence was not reached or singular fit issues persisted, we continued simplifying the random slopes of other factors until convergence was achieved.

### 2.2. Results

**Figure 1:**
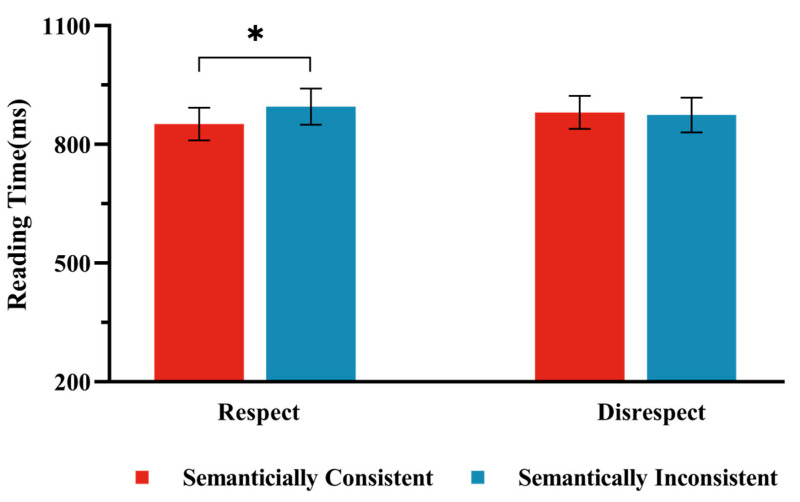
Reading Times in Experiment 1. The bar chart presents the results under two conditions: the “Respect” condition, where the pronoun “nin” was used, and the “Disrespect” condition, where the pronoun “ni” was used. Note: Error bars represent standard errors. * indicates a significance level of *p* < 0.05.

The average accuracy of participants’ reading comprehension was 91.07% (*SD* = 0.04, *ACC* range = 82.50–96.25%), indicating that all participants read the materials attentively. For the analysis of the reading time of the critical sentences, trials with reading times exceeding 3 standard deviations from the participant’s average were removed (Ju et al., 2024), resulting in the deletion of 3.85% of the data. The mean reading times for the four conditions in Experiment l are presented in Figure 1. As shown in Table 3, we found a significant interaction between Semantic Consistency and Respect Consistency (*t* (3450.99) = 2.25, *p* = 0.03); a simple effects analysis revealed that, under the Respect condition, there was a significant difference between the Semantically Consistent condition and the Semantically Inconsistent condition (*t* (47) = 2.62, *p* < 0.05), with reading times in the Semantically Inconsistent condition (*M* = 897, 95%CI [802, 992]) being significantly longer than those in the Semantically Consistent condition (*M* = 850, 95%CI [762, 937]). In contrast, under the Disrespect condition, no significant difference was observed between the Semantically Consistent condition and the Inconsistent condition (*t* (112) = 0.34, *p* = 0.73); there was no significant difference in reading times between the Semantically Incongruent condition and the Semantically Consistent condition (*M* = 883, 95%CI [794, 972]). The main effect of Respect Consistency was not significant (*t* (77.43) = −0.30, *p* = 0.76); there was no significant difference in reading times between the Respect condition (*M* = 873, 95%CI [785, 962]) and the Disrespect condition (*M* = 879, 95%CI [790, 968]). The main effect of Semantic Consistency was also not significant (*t* (65.22) = 0.96, *p* = 0.34); there was no significant difference in reading times between the Semantically Inconsistent condition (*M* = 886, 95%CI [793, 978]) and the Semantically Consistent condition (*M* = 866, 95%CI [781, 952]).

### 2.3. Discussion

The results of this study revealed a classic semantic integration effect under the Respect condition, where the reading time in the Semantic Inconsistent condition was longer than in the Semantic Consistent condition, which aligns with the previous research (Y. Shi et al., 2024; Wu et al., 2019; Lassonde & O’Brien, 2013). More importantly, this study found that, under the Respect condition, the semantic consistency effect was stronger compared to the effect observed under the Disrespect condition. These findings are consistent with previous studies (Jiang et al., 2013), further supporting the idea that the inappropriate use of the pronoun “ni” (you_[plain]_) significantly affects discourse semantic integration.

Note that previous studies have primarily focused on the online processing of sentences (Ji, 2021; Ji & Cai, 2022; Ji et al., 2022) and have not thoroughly explored the impact of respectful terms on discourse semantic integration. In contrast, this study demonstrates that under the Disrespect condition, the integration difference between the Semantic Consistent condition and the Semantic Inconsistent condition was diminished, which supports the assumptions of the global resource theory (MacKay et al., 2004). According to the global resource theory, attentional resources are limited, and the Disrespect condition demands more of the participants’ attention, making it harder for them to detect whether the semantics are consistent (MacKay et al., 2004). Notably, these results diverge from the predictions of the binding theory, which suggests that inappropriate words should facilitate semantic integration (Christianson et al., 2017). Contrary to this expectation, no significant difference was observed between the Semantically Consistent and Semantically Inconsistent conditions under the Disrespect condition in our study.

In summary, the results of Experiment 1 suggest that the inappropriate use of respectful pronouns significantly influences semantic integration during discourse integration.

## 3. Experiment 2

Experiment 1 examines the effect of Respect Consistency (“nin” (you_[respectful]_) vs. “ni” (you_[plain]_)) on semantic integration. It tests whether respectful pronouns facilitate semantic processing more than non-respectful ones. The pronouns “nin” (you_[respectful]_) and “ni” (you_[plain]_) are common and familiar terms, frequently used in daily conversations. It is perhaps this familiarity that enabled us to observe their influence. Experiment 2 shifts from pronouns to personal names (e.g., Zhu Laoshi [Teacher Zhu] vs. Zhu Wenyi [Zhu Wenyi]), investigating whether the personal names condition affects integration similarly to pronouns. Personal names are also commonly used in daily interactions. But as discussed in the review, one should not directly address people of higher status by their name (Kwon & Sturt, 2016). This raises the question: Could personal names lead to different results compared to pronouns? Unlike the familiar “nin” (you_[respectful]_) and “ni” (you_[plain]_), personal names used in the material are less familiar to participants. Could it be that the participants do not engage in much integration because they do not notice the condition setup? To test this issue, we designed Experiment 2 to explore whether the personal names condition, as a form of disrespect, produces different effects compared to the disrespectful “ni” (you_[plain]_) condition used in Experiment 1.

### 3.1. Methods

#### 3.1.1. Participants

Experiment 2 recruited 39 university and graduate students, aged 18–26 years (mean age = 21.45 ± 2.53 years), including 8 males and 31 females. All participants were undergraduate or graduate students at Renmin University of China, long-term residents of China, and self-reported native speakers of Chinese. They passed the Putonghua Proficiency Test and scored above 120 on the Chinese section of the National College Entrance Examination, ensuring high Mandarin proficiency and minimizing dialectal influences. All were right-handed, had normal or corrected-to-normal vision, and had no history of reading disabilities or psychiatric disorders. The experiment was approved by the Academic Ethics Committee of the Department of Psychology at Renmin University of China. Participants signed an informed consent form before the experiment and received compensation upon completion.

#### 3.1.2. Materials, Procedure, and Data Analysis

The 80 sets of experimental sentences from Experiment 1 were modified for use in Experiment 2. Specifically, in the third clause, the respectful pronouns “nin” (you_[respectful]_) or “ni” (you_[plain]_) were replaced with a title (occupational title, e.g., Teacher Zhu) or name (e.g., Zhu Wenyi) (see Table 4). A separate group of 16 participants was recruited to evaluate the modified materials. We calculated the scores for the two dimensions under different conditions. For Respectful Consistency, the scores were as follows: Respect (*M* = 6.78, *SD* = 0.23), Disrespect (*M* = 2.22, *SD* = 0.67). For Semantic Consistency, the scores were Semantically Consistent (*M* = 5.98, *SD* = 0.61), Semantically Inconsistent (*M* = 1.95, *SD* = 0.69). A *t*-test was conducted to analyze the evaluation results, which revealed a significant difference in Respect Consistency, *t* (79) = 114.71, *p* < 0.01, indicating that the manipulation of respectful terms met the experimental requirements. Additionally, a significant difference in Semantic Consistency was observed, *t* (79) = 47.00, *p* < 0.01, confirming that the manipulation of semantics also met the experimental standards.

As in Experiment 1, the 80 sets of sentences were divided into 4 versions, with 80 filler sentences added to each version. The procedure and data analysis methods were the same as those used in Experiment 1.

### 3.2. Results

**Figure 2:**
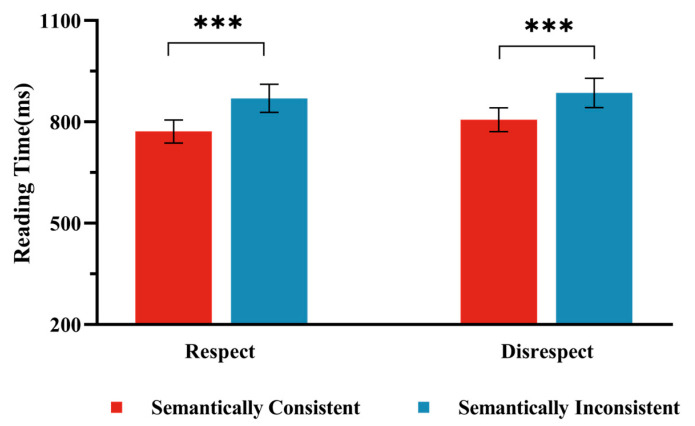
Reading Times in Experiment 2. The bar chart presents the results under two conditions: the “Respect” condition, where an appropriately respectful form of address was used, and the “Disrespect” condition, where a personal name was used. Note: Error bars represent standard errors. *** indicates a significance level of *p* < 0.001.

The average accuracy of participants in the reading comprehension task was 92.62% (*SD* = 0.03, *ACC* range = 82.89–97.50%), indicating that participants read the materials attentively. For the analysis of the reading times for key sentences, trials with reading times exceeding 3 *SD*s from the participant’s mean were excluded (Ju et al., 2024), resulting in the removal of 2.31% of the data. The mean reading times for the four conditions in Experiment l are presented in Figure 2. As shown in Table 5, we found a significant main effect of Semantic Consistency (*t* (64.50) = 4.67, *p* < 0.01), with reading times in the Semantically Inconsistent condition (*M* = 880, 95%CI [793, 967]) being significantly longer than those in the Semantically Consistent condition (*M* = 789, 95% CI [718, 860]). However, the main effect of Respect Consistency was not significant ( *t* (76.40) = −1.70, *p* = 0.09), with reading times similar between the Respect condition (*M* = 821, 95%CI [743, 900]) and the Disrespect condition (*M* = 848, 95%CI [769, 926]). The interaction between Respect Consistency and Semantic Consistency was also non-significant (*t* (76.75) = 0.61, *p* = 0.54); under the Respect condition, there was no significant difference between the Semantically Consistent condition and the Semantically Inconsistent condition (*t* (2929) = −6.40, *p* < 0.01), with reading times in the Semantically Inconsistent condition (*M* = 871, 95%CI [792, 950]) being significantly longer than those in the Semantically Consistent condition (*M* = 771 95%CI [692, 850]). Under the Disrespect condition, no significant difference was observed between the Semantically Consistent condition and the Inconsistent condition (*t* (2930) = −5.25, *p* < 0.01), with reading times in the Semantically Inconsistent condition (*M* = 888, 95%CI [808, 967]) being significantly longer than those in the Semantically Consistent condition (*M* = 806, 95%CI [727, 885]).

### 3.3. Discussion

The results of Experiment 2 revealed the classic semantic integration effect, where reading times were longer for the Semantically Inconsistent conditions compared to the Semantically Consistent conditions, which is consistent with the previous research (Y. Shi et al., 2024; Wu et al., 2019; Lassonde & O’Brien, 2013). In contrast to Experiment 1, Experiment 2 used personal names and introduced calling someone of higher status directly by their personal names as a disrespect condition (personal names). The results revealed a noteworthy effect of semantics only, without any significant interaction effects.

We speculate that this difference may be due to participants not noticing the name-based conditions. The reading comprehension task required participants to make judgments based on the semantics of the text, and the presence of names was unrelated to the task itself. As a result, participants likely paid little attention to the personal names and instead focused on the semantic content to ensure accurate comprehension judgments.

## 4. Experiment 3

Although Experiment 2 did not reveal a significant interaction between Semantic Consistency and Respect Consistency, a trend similar to that observed in Experiment 1 was noted: the semantic consistency effect (the difference between the semantic inconsistent and consistent conditions) was smaller under the Disrespect condition than under the Respect condition. To further investigate the potential influence of personal names on discourse semantic integration, Experiment 3 was designed. It is likely that the lack of interaction in Experiment 2 could be due to participants not noticing the personal names condition. Therefore, Experiment 3 incorporates an appropriateness judgment task to examine whether explicitly evaluating respect enhances the interaction between Semantic Consistency and Respect Consistency, directing attentional resources toward appropriateness.

### 4.1. Methods

#### 4.1.1. Participants

In total, forty college students participated in Experiment 3, ranging in age from 18 to 24 years (mean age = 19.95 ± 1.66 years), including 12 males and 28 females. All participants were undergraduate or graduate students at Renmin University of China, long-term residents of China, and self-reported native speakers of Chinese. They passed the Putonghua Proficiency Test and scored above 120 on the Chinese section of the National College Entrance Examination, ensuring high Mandarin proficiency and minimizing dialectal influences. All were right-handed, had normal or corrected-to-normal vision, and had no history of reading disabilities or psychiatric disorders. This study was approved by the Academic Ethics Committee of the Department of Psychology at Renmin University of China. Prior to the experiment, all participants provided informed consent, and they received compensation following the completion of this study.

#### 4.1.2. Procedure, and Data Analysis

The experimental materials were identical to those used in Experiment 2; however, the task content and occurrence probabilities differed. Following the reading of each passage, participants were asked to judge its appropriateness within 3 s by answering the question, “e.g., Is the current passage appropriate?”. The data analysis methods employed were consistent with those used in Experiments 1 and 2.

### 4.2. Results

Participants judged the appropriateness of the passages. Specifically, for the Disrespect–Semantically Inconsistent condition, 20% were judged as appropriate; for the Respect–Semantically Inconsistent condition, 26% were judged as appropriate; for the Disrespect–Semantically Consistent condition, 53% were judged as appropriate; and for the Respect–Semantically Consistent condition, 80% were judged as appropriate. These results indicate that the participants not only paid attention to the semantic appropriateness but also noticed the appropriateness of respectfulness.

**Figure 3:**
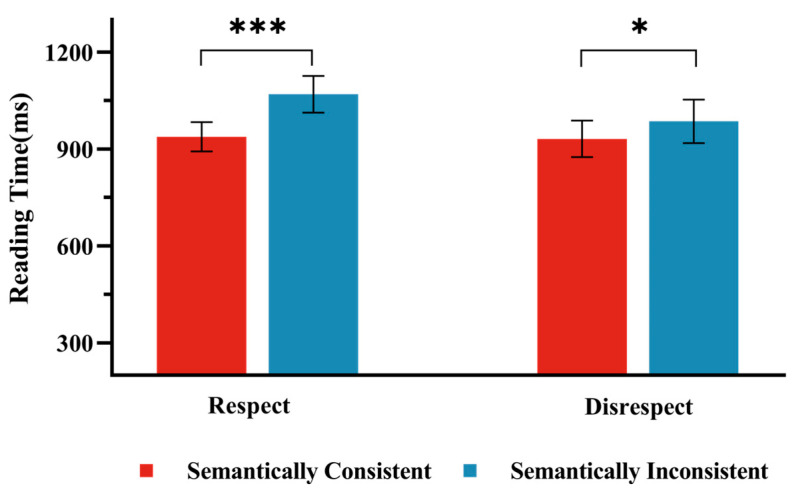
Reading Times in Experiment 3. The bar chart presents the results under two conditions: the “Respect” condition, where an appropriately respectful form of address was used, and the “Disrespect” condition, where a personal name was used. Note: Error bars represent standard errors. *** indicates a significance level of *p* < 0.001, * indicates a significance level of *p* < 0.05.

Analysis of key sentence reading times first excluded trials exceeding 3 standard deviations from participants’ mean times (Ju et al., 2024), resulting in the removal of 2.16% of the data. The mean reading times for the four conditions in Experiment l are presented in Figure 3. As shown in Table 6, a significant main effect of Semantic Consistency was observed (*t* (3012.20) = 5.36, *p* < 0.01). Reading times in the Semantically Inconsistent condition (M = 1027, 95%CI [915, 1138]) were significantly longer than those in the Semantically Consistent condition (M = 933, 95%CI [822, 1044]). A significant main effect of Respect Consistency was also found (*t* (3012.00) = 2.57, *p* = 0.01), with reading times in the Disrespect condition (M = 1002, 95%CI [891, 1113]) being significantly longer than those in the Respect condition (M = 958, 95%CI [847, 1069]). Furthermore, a significant interaction between Semantic Consistency and Respect Consistency was observed (*t* (3011.88) = 2.20, *p* = 0.03). Simple effects analysis revealed a significant difference between the Semantically Inconsistent conditions and the Semantically Consistent conditions under the Respect condition (*t* (3012.00) = −5.34, *p* < 0.01), with reading times in the Semantically Inconsistent condition (M = 985, 95%CI [872, 1099]) being significantly longer than those in the Semantically Consistent condition (M = 930, 95% CI [817, 1044]). Under the Disrespect condition, the difference between the Semantically Inconsistent conditions and the Semantically Consistent conditions was still significant but smaller (*t* (3012.00) = −2.24, *p* = 0.03), with reading times in the Semantically Incongruent condition (M = 1068, 95%CI [955, 1182]) being significantly longer than those in the Semantically Consistent condition (M = 937, 95% CI [823, 1050]).

### 4.3. Discussion

Experiment 3 employed a more explicit appropriateness judgment task than Experiment 2 to further investigate the impact of respectful terms on semantic integration. The results once again demonstrated the classic semantic integration effect, where reading times for the Semantic Inconsistent conditions were longer than for the Semantically Consistent conditions, consistent with prior research (Y. Shi et al., 2024; Wu et al., 2019; Lassonde & O’Brien, 2013). Additionally, Experiment 3 revealed a significant interaction between Respect Consistency and Semantic Consistency. In the Respect condition, reading times for the Semantically Inconsistent conditions were longer than for the Semantically Consistent conditions. In contrast, in the Disrespect condition, a smaller significant difference was found between the Semantically Consistent conditions and the Semantically Inconsistent conditions. These results further support the global resource theory, which suggests that attentional resources are limited (MacKay et al., 2004). The Disrespect condition, characterized by the use of personal names, likely attracted more participant attention, thereby diminishing their focus on the overall semantic integration.

## 5. General Discussion

This study conducted three experiments to examine how semantics are integrated during discourse comprehension, manipulating both Respect Consistency and Semantic Consistency. Across all three experiments, the classic semantic integration effect was observed: reading times for the Semantically Inconsistent conditions were longer than for the Semantically Consistent conditions. More importantly, the findings revealed that the semantic integration effect was influenced by the use of respectful terms. In both Experiments 1 and 3, a significant interaction between Respect Consistency and Semantic Consistency was observed. Experiment 2 exhibited a similar trend, where the difference between the Semantic Consistent conditions and the Semantic Inconsistent conditions was smaller under the Disrespect condition compared to the Respect condition. These results suggest that inappropriate respectful terms in discourse hinder semantic integration.

In this study, semantic inconsistency were introduced in S1 and S4, resulting in a typical semantic integration effect, where integration times for the Semantically Consistent conditions were longer than for the Semantically Consistent conditions. The results of this study are consistent with those from semantic violation paradigms, which show that semantically inconsistent conditions elicit larger N400 components compared to semantically consistent conditions (Y. Shi et al., 2024; Chang et al., 2020; X. Yang et al., 2020; Wu et al., 2019; Wang et al., 2011; Lassonde & O’Brien, 2013). In line with these studies, our results further demonstrate that semantic inconsistency impedes sentence integration, thereby validating the effectiveness of the semantic manipulations in the materials used.

More importantly, the semantic integration effect observed in this study was influenced by the use of respectful terms. Regardless of whether respectful pronouns (Experiment 1) or personal name (Experiment 3) were used, the interaction between Respect Consistency and Semantic Consistency was significant. Under the Respect condition, a clear difference was observed between Semantically Consistent conditions and Semantically Inconsistent conditions, whereas under the Disrespect condition, this difference was non-significant or less significant compared to the Respect condition. This suggests that in the Respect condition, the classic semantic integration effect remains intact, with respect not disrupting semantic integration. However, in the Disrespect condition, not using respectful pronouns to address people of higher social status attracted more participants’ attention and consumed additional cognitive resources, preventing participants from noticing semantic inconsistency. These findings further support the global resource theory (MacKay et al., 2004). According to this theory, Disrespect conditions draw participants’ attentional resources, thereby reducing the resources available for semantic integration.

The role of personal name varied based on task settings. Experiments 2 and 3 used identical materials to examine the impact of personal name on discourse integration, differing only in task design. Experiment 2 employed a reading comprehension task (implicit task), while Experiment 3 used an appropriateness judgment task (explicit task). The results differed between the two experiments. The reading comprehension task in Experiment 2 focused on discourse semantics, and the exclusive appearance of the semantic integration effect suggests that participants primarily attended to semantic integration. Prior research has shown that readers integrate background information in discourse. In everyday situations, people often skim texts for general meaning rather than reading closely (Masson, 1982). Participants in Experiment 2 likely judged sentences based on semantics, without attending closely to personal names. In contrast, Experiment 3, which employed the appropriateness judgment task, encouraged participants to actively focus on appropriateness, thereby a significant interaction between Respect Consistency and Semantic Consistency was observed. The Disrespect condition attracted more participant attention, leaving insufficient attentional resources to focus on semantic integration. As a result, no significant difference was found in Semantic Consistency under the Disrespect condition. Comparing the results of the two experiments, we infer that the effect of personal name is more evident under explicit task conditions, where participants can effectively detect the Disrespect condition. This is also in line with the previous research which found that task type influences processing (White et al., 2015).

There are differences in the processing of respectful pronouns and personal name. In Experiment 1, using respectful pronouns, we easily observed the interaction between Respect Consistency and Semantic Consistency. However, in Experiments 2 and 3, which used personal name, the emergence of this interaction was influenced by the task type. In the explicit appropriateness judgment task, we observed the interaction between respect and semantics. We speculate that the familiarity of the pronouns “ni” (you_[plain]_) and “nin” (you_[respectful]_) plays a role, as they are more commonly used and familiar.

This study demonstrates that the Respect and Disrespect conditions hinder semantic integration in controlled settings, yet its generalizability to real-world contexts remains unclear, necessitating further research. First, although all participants were native Chinese speakers, individual differences—such as social sensitivity, age, education, occupation, and dialect—may affect perceptions of respectful terms, with older or hierarchically experienced individuals potentially showing greater sensitivity. Moreover, the politeness system in Chinese differs from more complex honorific systems (e.g., Japanese or Korean), limiting cross-linguistic applicability. Future studies should explore these individual and cross-linguistic variations. Second, when we used personal names to manipulate respectful usage, the interaction between Semantic Consistency and Respect Consistency emerged only in the explicit judgment task (Experiment 3), not the implicit reading comprehension task (Experiment 2), suggesting task type influences cognitive resource allocation. Further research should investigate this distinction, potentially using advanced methods like eye-tracking or EEG for greater precision.

This study not only extends the application of the global resource theory to the domain of respectful term usage but also provides novel insights by demonstrating how sociocultural cues, such as appropriate respectfulness and personal names, modulate semantic integration in discourse—a dimension underexplored in prior research focused predominantly on lexical-semantic or single-sentence processing (e.g., Ji, 2021; Y. Shi et al., 2024). Beyond its theoretical contributions, these findings have practical implications for cross-cultural communication, where the appropriate use of respectful address terms is critical. For instance, in multilingual or intercultural settings—such as business negotiations, diplomatic exchanges, or language education—misalignment in respectful language use could disrupt comprehension and strain interpersonal relations, particularly when translating between languages with differing honorific systems. By illuminating how disrespect hinders semantic integration, this study emphasizes the need for cultural sensitivity in communication training, ensuring that social nuances are preserved to facilitate effective discourse across diverse linguistic contexts.

In conclusion, the previous research on respectful terms has primarily focused on pronouns like “nin” (you_[respectful]_) and “ni” (you_[plain]_) in short sentences (1–2 clauses), often examining the impact of inappropriate pronoun use (Jiang et al., 2013; Jiang & Zhou, 2015; Ji, 2021; Ji et al., 2022; Ji & Cai, 2022; Ji & Ji, 2023). This study extends the prior research by broadening the scope from respectful pronouns (“nin” (you_[respectful]_) and “ni” (you_[plain]_)) to personal name (appropriate respectfulness vs. personal names) and examining the influence of respectful terms on semantic integration at discourse level. The result indicates that the use of respectful terms significantly impacts semantic integration, with different respectful terms influencing this integration in distinct ways. These findings provide important insights into the role of respectful terms on the integration of discourse.

## Figures and Tables

**Table 1:** Predictions for Experiments 1–3. Based on the global resource theory and the binding theory. RT represents reading time in this table.

	Global Resource Theory	Binding Theory
Experiment 1	Respect condition: RT for Semantically Inconsistent > RT for Semantically Consistent. Disrespect condition: no differences/smaller differences compared to the Respect condition.	Respect condition: RT for Semantically Inconsistent > RT for Semantically Consistent. Disrespect condition: larger differences compared to the Respect condition.
Experiment 2	We expect to obtain similar results to those in Experiment 1.	We expect to obtain similar results to those in Experiment 1.
Experiment 3	We anticipate that the results may resemble those of Experiment 1.	We anticipate that the results may resemble those of Experiment 1.

**Table 2:** Design and stimulus examples for all four critical conditions are given in Chinese, with English glosses and translations.

Sentence Type	Example
Respect, Semantically Consistent	7岁的小明关上门，/他对老师说：/“这是您要的材料。”/然后继续写作业。/小明非常懂事。
	Gloss: Seven-year-old Xiaoming closed the door,/He said to the teacher: /“This is you_[respectful]_ requested material.”/Then continued doing homework./Xiaoming is very well-behaved.
	Translation: Seven-year-old Xiaoming closed the door,/said to the teacher,/“This is the material you_[respectful]_ asked for,”/and then continued doing his homework./Xiaoming is very considerate.
Respect, Semantically Inconsistent	30岁的小明关上门，/他对老师说：/“这是您要的材料。”/然后继续写作业。/小明非常懂事。
	Gloss: Thirty-year-old Xiaoming closed the door,/He said to the teacher:/“This is you_[respectful]_ requested material.”/Then continued doing homework./Xiaoming is very well-behaved.
	Translation: Thirty-year-old Xiaoming closed the door,/said to the teacher,/“This is the material you_[respectful]_ asked for,”/and then continued doing his homework./Xiaoming is very considerate.
Disrespect, Semantically Consistent	7岁的小明关上门，/他对老师说：/“这是你要的材料。”/然后继续写作业。/小明非常懂事。
	Gloss: Seven-year-old Xiaoming closed the door,/He said to the teacher:/“This is you_[plain]_ requested material.”/Then continued doing homework./Xiaoming is very well-behaved.
	Translation: Seven-year-old Xiaoming closed the door,/said to the teacher,/“This is the material you_[plain]_ asked for,”/and then continued doing his homework./Xiaoming is very considerate.
Disrespect, Semantically Inconsistent	30岁的小明关上门，/他对老师说：/“这是你要的材料。”/然后继续写作业。/小明非常懂事。
	Gloss: Thirty-year-old Xiaoming closed the door,/He said to the teacher:/“This is you_[plain]_ requested material.”/Then continued doing homework./Xiaoming is very well-behaved.
	Translation: Thirty-year-old Xiaoming closed the door,/said to the teacher,/“This is the material you_[plain]_ asked for,”/and then continued doing his homework./Xiaoming is very considerate.

**Table 3:** Model parameters for the best-fitting linear mixed-effects model of RT (reading time) for Experiment 1, with fixed factors of respect, consistency, and their interactions.

Model Parameters	β	SE	df	*t*	*p*	95%CI
Respect	−0.01	0.02	77.43	−0.30	0.76	[−0.045, 0.033]
Semantic Consistency	0.02	0.02	65.22	0.96	0.34	[−0.021, 0.061]
Respect * Semantic Consistency	0.03	0.03	3450.99	2.25	0.03	[0.004, 0.053]

RT = reading time, * denotes an interaction between the two variables.

**Table 4:** Design and stimulus examples for all four critical conditions are given in Chinese, with English glosses and translations.

Sentence Type	Sentence Type
Respect, Semantically Consistent	7岁的小明关上门，/他对老师说：/“朱老师，这是相关的材料。”/然后继续写作业。/小明非常懂事。
	Gloss: Seven-year-old Xiaoming closed the door,/He said to the teacher:/“Teacher Zhu_[respectful]_, this is the relevant material,”/Then continued writing homework./Xiaoming is very well-behaved.
	Translation: Seven-year-old Xiaoming closed the door,/said to the teacher:/“Teacher Zhu_[respectful]_, this is the relevant material,”/and then continued doing his homework./Xiaoming is very considerate.
Respect, Semantically Inconsistent	30岁的小明关上门，/他对老师说：/“朱老师，这是相关的材料。”/然后继续写作业。/小明非常懂事。
	Gloss: Thirty-year-old Xiaoming closed the door,/He said to the teacher:/“Teacher Zhu_[respectful]_, this is the relevant material,”/Then continued writing homework./Xiaoming is very well-behaved.
	Translation: Thirty-year-old Xiaoming closed the door,/said to the teacher,/“Teacher Zhu_[respectful]_, This is the material you_[respectful]_ asked for,”/and then continued doing his homework./Xiaoming is very considerate.
Disrespect, Semantically Consistent	7岁的小明关上门，/他对老师说：/“朱文轶，这是相关的材料。”/然后继续写作业。/小明非常懂事。
	Gloss: Seven-year-old Xiaoming closed the door,/He said to the teacher:/“Zhu Wenyi_[plain]_, this is the relevant material,”/Then continued writing homework./Xiaoming is very well-behaved.
	Translation: Seven-year-old Xiaoming closed the door,/said to the teacher,/“Zhu Wenyi_[plain]_, This is the material you_[respectful]_ asked for,”/and then continued doing his homework./Xiaoming is very considerate.
Disrespect, Semantically Inconsistent	30岁的小明关上门，/他对老师说：/“朱文轶，这是相关的材料。”/然后继续写作业。/小明非常懂事。
	Gloss: Thirty-year-old Xiaoming closed the door,/ He said to the teacher:/“Zhu Wenyi_[plain]_, this is the relevant material,”/Then continued writing homework./Xiaoming is very well-behaved.
	Translation: Thirty-year-old Xiaoming closed the door,/said to the teacher,/“Zhu Wenyi_[plain]_, this is the relevant material,”/and then continued doing his homework./Xiaoming is very considerate.

**Table 5:** Model parameters for the best-fitting linear mixed-effects model of RT for Experiment 2, with fixed factors of respect, consistency, and their interactions.

Model Parameters	β	SE	df	*t*	*p*	95%CI
Respect	−0.03	0.02	74.40	−1.70	0.09	[−0.073, 0.006]
Semantic Consistency	0.12	0.03	64.50	4.67	<0.01	[0.067, 0.167]
Respect * Semantic Consistency	0.01	0.02	76.75	0.61	0.54	[−0.023, 0.044]

RT = reading time, * denotes an interaction between the two variables.

**Table 6:** Model parameters for the best-fitting linear mixed-effects model of RT (reading time) for Experiment 3, with fixed factors of respect, consistency, and their interactions.

Model Parameters	β	SE	df	*t*	*p*	95%CI
Respect	0.04	0.02	3012.00	2.57	0.01	[0.009, 0.066]
Semantic Consistency	0.08	0.02	3012.20	5.36	<0.01	[0.049, 0.106]
Respect * Semantic Consistency	0.03	0.02	3011.88	2.20	0.03	[0.003, 0.061]

RT = reading time, * denotes an interaction between the two variables.

## Data Availability

The raw data supporting the conclusions of this article will be made available by the authors on request.
